# Supplementary material for: Unveiling the Bioactive Potential of the Invasive Jellyfish Phyllorhiza punctata Through Integrative Transcriptomic and Proteomic Analyses
Source: Biomolecules. 2025 Aug 4;15(8):1121. doi: 10.3390/biom15081121 (PMC12383608; doi:10.3390/biom15081121)
Supplement: Supplementary file 1 [file biomolecules-15-01121-s001.zip › Suplementary Material/FileS3/PEPFOLD4/XP_065065168_4256/clusters.html]

 PEP-FOLD clusters


Download archive of all models  here   

|  |  |  |
| --- | --- | --- |
| # | file | sOPEP |
| # cluster 1 - Size: 44 |  |  |
|  | XP\_065065168\_4256-model1 | -15.5863 |
|  | XP\_065065168\_4256-model1.1 | -15.5863 |
|  | XP\_065065168\_4256-model1.2 | -15.5834 |
|  | XP\_065065168\_4256-model1.3 | -15.499 |
|  | XP\_065065168\_4256-model1.4 | -15.4351 |
|  | XP\_065065168\_4256-model1.5 | -15.3019 |
|  | XP\_065065168\_4256-model1.6 | -15.2826 |
|  | XP\_065065168\_4256-model1.7 | -15.2392 |
|  | XP\_065065168\_4256-model1.8 | -15.2392 |
|  | XP\_065065168\_4256-model1.9 | -15.2189 |
|  | XP\_065065168\_4256-model1.10 | -15.2189 |
|  | XP\_065065168\_4256-model1.11 | -15.2178 |
|  | XP\_065065168\_4256-model1.12 | -15.1925 |
|  | XP\_065065168\_4256-model1.13 | -15.1536 |
|  | XP\_065065168\_4256-model1.14 | -15.1441 |
|  | XP\_065065168\_4256-model1.15 | -15.122 |
|  | XP\_065065168\_4256-model1.16 | -15.0828 |
|  | XP\_065065168\_4256-model1.17 | -15.0649 |
|  | XP\_065065168\_4256-model1.18 | -15.0566 |
|  | XP\_065065168\_4256-model1.19 | -15.0356 |
|  | XP\_065065168\_4256-model1.20 | -15.0286 |
|  | XP\_065065168\_4256-model1.21 | -15.0107 |
|  | XP\_065065168\_4256-model1.22 | -14.9777 |
|  | XP\_065065168\_4256-model1.23 | -14.963 |
|  | XP\_065065168\_4256-model1.24 | -14.963 |
|  | XP\_065065168\_4256-model1.25 | -14.963 |
|  | XP\_065065168\_4256-model1.26 | -14.9337 |
|  | XP\_065065168\_4256-model1.27 | -14.9333 |
|  | XP\_065065168\_4256-model1.28 | -14.9333 |
|  | XP\_065065168\_4256-model1.29 | -14.9333 |
|  | XP\_065065168\_4256-model1.30 | -14.9086 |
|  | XP\_065065168\_4256-model1.31 | -14.9047 |
|  | XP\_065065168\_4256-model1.32 | -14.9047 |
|  | XP\_065065168\_4256-model1.33 | -14.8787 |
|  | XP\_065065168\_4256-model1.34 | -14.8668 |
|  | XP\_065065168\_4256-model1.35 | -14.855 |
|  | XP\_065065168\_4256-model1.36 | -14.8499 |
|  | XP\_065065168\_4256-model1.37 | -14.8499 |
|  | XP\_065065168\_4256-model1.38 | -14.8499 |
|  | XP\_065065168\_4256-model1.39 | -14.8329 |
|  | XP\_065065168\_4256-model1.40 | -14.791 |
|  | XP\_065065168\_4256-model1.41 | -14.7696 |
|  | XP\_065065168\_4256-model1.42 | -14.5411 |
|  | XP\_065065168\_4256-model1.43 | -14.5061 |
| # cluster 2 - Size: 20 |  |  |
|  | XP\_065065168\_4256-model2 | -15.5354 |
|  | XP\_065065168\_4256-model2.1 | -15.3976 |
|  | XP\_065065168\_4256-model2.2 | -15.3431 |
|  | XP\_065065168\_4256-model2.3 | -15.2734 |
|  | XP\_065065168\_4256-model2.4 | -15.1543 |
|  | XP\_065065168\_4256-model2.5 | -15.1141 |
|  | XP\_065065168\_4256-model2.6 | -15.1001 |
|  | XP\_065065168\_4256-model2.7 | -15.0815 |
|  | XP\_065065168\_4256-model2.8 | -15.0608 |
|  | XP\_065065168\_4256-model2.9 | -14.9263 |
|  | XP\_065065168\_4256-model2.10 | -14.9199 |
|  | XP\_065065168\_4256-model2.11 | -14.8436 |
|  | XP\_065065168\_4256-model2.12 | -14.7907 |
|  | XP\_065065168\_4256-model2.13 | -14.7486 |
|  | XP\_065065168\_4256-model2.14 | -14.7477 |
|  | XP\_065065168\_4256-model2.15 | -14.7196 |
|  | XP\_065065168\_4256-model2.16 | -14.6714 |
|  | XP\_065065168\_4256-model2.17 | -14.6356 |
|  | XP\_065065168\_4256-model2.18 | -14.2748 |
|  | XP\_065065168\_4256-model2.19 | -14.2362 |
| # cluster 3 - Size: 10 |  |  |
|  | XP\_065065168\_4256-model3 | -15.4083 |
|  | XP\_065065168\_4256-model3.1 | -15.3646 |
|  | XP\_065065168\_4256-model3.2 | -15.2042 |
|  | XP\_065065168\_4256-model3.3 | -14.9038 |
|  | XP\_065065168\_4256-model3.4 | -14.8177 |
|  | XP\_065065168\_4256-model3.5 | -14.7261 |
|  | XP\_065065168\_4256-model3.6 | -14.715 |
|  | XP\_065065168\_4256-model3.7 | -14.6862 |
|  | XP\_065065168\_4256-model3.8 | -14.325 |
|  | XP\_065065168\_4256-model3.9 | -14.0142 |
| # cluster 4 - Size: 4 |  |  |
|  | XP\_065065168\_4256-model4 | -14.8855 |
|  | XP\_065065168\_4256-model4.1 | -14.8061 |
|  | XP\_065065168\_4256-model4.2 | -14.5615 |
|  | XP\_065065168\_4256-model4.3 | -14.4947 |
| # cluster 5 - Size: 6 |  |  |
|  | XP\_065065168\_4256-model5 | -11.2385 |
|  | XP\_065065168\_4256-model5.1 | -10.9697 |
|  | XP\_065065168\_4256-model5.2 | -10.9187 |
|  | XP\_065065168\_4256-model5.3 | -10.7644 |
|  | XP\_065065168\_4256-model5.4 | -10.5458 |
|  | XP\_065065168\_4256-model5.5 | -10.4475 |
| # cluster 6 - Size: 1 |  |  |
|  | XP\_065065168\_4256-model6 | -11.0128 |
| # cluster 7 - Size: 7 |  |  |
|  | XP\_065065168\_4256-model7 | -10.8753 |
|  | XP\_065065168\_4256-model7.1 | -10.8468 |
|  | XP\_065065168\_4256-model7.2 | -10.7418 |
|  | XP\_065065168\_4256-model7.3 | -10.5879 |
|  | XP\_065065168\_4256-model7.4 | -10.4518 |
|  | XP\_065065168\_4256-model7.5 | -10.4345 |
|  | XP\_065065168\_4256-model7.6 | -10.4124 |
| # cluster 8 - Size: 3 |  |  |
|  | XP\_065065168\_4256-model8 | -10.7462 |
|  | XP\_065065168\_4256-model8.1 | -10.6991 |
|  | XP\_065065168\_4256-model8.2 | -10.558 |
| # cluster 9 - Size: 1 |  |  |
|  | XP\_065065168\_4256-model9 | -10.5251 |
| # cluster 10 - Size: 1 |  |  |
|  | XP\_065065168\_4256-model10 | -10.4099 |
| # cluster 11 - Size: 2 |  |  |
|  | XP\_065065168\_4256-model11 | -10.3696 |
|  | XP\_065065168\_4256-model11.1 | -10.2972 |
| # cluster 12 - Size: 1 |  |  |
|  | XP\_065065168\_4256-model12 | -10.2407 |
|  |  |  |
